# Identifying Facilitators and Barriers to Increasing COVID-19 Vaccination and Trial Participation in Vaccinated Vietnamese Americans

**DOI:** 10.1089/heq.2022.0032

**Published:** 2022-06-27

**Authors:** Celine Nguyen, Lauren Gilbert, Jannette Diep, Bich-May Nguyen

**Affiliations:** ^1^ Boat People SOS Houston, Houston, Texas, USA.; ^2^ Department of Health Systems and Population Health Sciences, University of Houston Tilman J. Fertitta Family College of Medicine, Houston, Texas, USA.

**Keywords:** health disparities, immigrant health, minority health, public health, qualitative research

## Abstract

**Background:**

Preventing morbidity and mortality from COVID-19 requires reaching diverse communities.

**Purpose:**

To identify facilitators and barriers to COVID-19 immunization and COVID-19 clinical trial participation in the vaccinated Vietnamese American population in Houston, TX.

**Methods:**

Community-based qualitative study using focus groups and key informant interviews.

**Results:**

Themes that emerged included culturally appropriate language, generational differences, and a collectivist approach.

**Conclusion:**

Promoting science-based information through trusted messengers, improving awareness and access, and illuminating benefits to the community could increase the uptake of COVID-19 vaccines and volunteering for therapeutic trials among Vietnamese Americans.

## Introduction

In the United States, the COVID-19 pandemic disproportionately impacted people of color, including Asian Americans, Native Hawaiians, and Pacific Islanders (AANHPI). While the AANHPI COVID-19 death rate is lower than its population share, the overall data were less commonly reported, AANHPI are often classified as “other,” and data usually are not disaggregated, underestimating disparities in and within the AANHPI population.^1,2^ In some areas that reported disaggregated data, Vietnamese Americans were overrepresented in coronavirus cases.^3,4^

To prevent severe illness and deaths from COVID-19, it is essential to reach diverse communities to raise awareness about measures such as COVID-19 vaccination and participation in clinical therapeutic trials. Some surveys have found that AANHPI are willing to receive COVID-19 vaccines, but they may have limited survey language accessibility, did not disaggregate data, or did not investigate specific reasons.^5,6^ Flores et al. found that 34% of vaccine trials from 2011 to 2020 reported ethnicity and did not disaggregate AANHPI data beyond Hawaiian, Pacific Islander, and Alaska Native.^7^

Our purpose was to identify facilitators and barriers to COVID-19 immunization and clinical trial participation in the Vietnamese American population.

## Materials and Methods

We conducted focus groups and key informant interviews between August to October 2021 using a community-engaged approach. Boat People SOS Houston (BPSOSH) used a convenience sampling strategy to recruit Vietnamese American adults in the Houston area who could choose English or Vietnamese for the survey, focus groups, and interviews. For key informant interviews, BPSOSH recruited faith leaders, health care workers, and small business owners for broad community perspectives. After obtaining informed consent from participants, facilitators and interviewers audio-recorded the sessions following a guide with open-ended questions, demonstrated in Tables 1 and 2.

**Table 1.** Focus Group Interview Guide

Vaccinated Groups Part 1: Group discussion
1. Tell me about how the COVID-19 pandemic has impacted your community
2. Where do you get your information regarding COVID-19 and the vaccines? a. Who are the trusted sources? Who are you wary of? b. What other information do you want to know about COVID-19 or the vaccine?
3. Did you have any concerns before or after receiving your vaccine? a. How did you overcome those concerns?
4. What reasons did you have for receiving the vaccine? a. Personal family b. Employment requirement/time off?
Now we are going to ask you some questions about COVID-19 clinical trials. *A clinical trial is a kind of research study. Clinical trials study if treatments or vaccines are safe for people and if they work like they are supposed to. Right now, clinical trials are being done across the U.S. to see if new treatments and vaccines for COVID-19 work to keep people healthy.*
5. Tell me about your experiences with clinical trials for the COVID-19 vaccine.
a. Have you ever participated?
b. Are you aware of anyone in your community participating in a clinical trial for the COVID-19 vaccine?
6. Are people in the community open/willing to participate in clinical trials? c. Why or why not?
Part 2: Ad review with PowerPoint
Focus group—ads
Now we are going to look at some sample informational ads. We are going to show you six ads for the COVID-19 vaccine. We will spend several minutes asking for your feedback on each one. For each ad,
1. How easy it is to understand?
2. Is this how you would say it to your family?
3. How appealing is this ad?
Unvaccinated Groups
Part 1: Group discussion
1. Tell me about how the COVID-19 pandemic has impacted your community
2. Where do you get your information regarding COVID-19 and the vaccines? a. Who are the trusted sources? Who are you wary of? b. What other information do you want to know about COVID-19 or the vaccine?
3. What reasons do you have for NOT receiving the vaccine? a. Concerns/hesitations/denials? b. Access issues? Do you know where to get it? c. Time off?
Now we are going to ask you some questions about COVID-19 clinical trials. *A clinical trial is a kind of research study. Clinical trials study if treatments or vaccines are safe for people and if they work like they are supposed to. Right now, clinical trials are being done across the U.S. to see if new treatments and vaccines for COVID-19 work to keep people healthy.*
4. Tell me about your experiences with clinical trials for the COVID-19 vaccine. d. Have you ever participated? e. Are you aware of anyone in your community participating in a clinical trial for the COVID-19 vaccine?
5. Are people in the community open/willing to participate in clinical trials? f. Why or why not?
Part 2: Ad review with PowerPoint
Focus group—ads
Now we are going to look at some sample informational ads. We are going to show you six ads for the COVID-19 vaccine. We will spend several minutes asking for your feedback on each one. For each ad, 1. How easy it is to understand?
2. Is this how you would say it to your family?
How appealing is this ad?

**Table 2.** Key Informant Interview Guide

1. Tell me about your role in the Vietnamese community
2. Tell me about how the COVID-19 pandemic has impacted your community. a. What have the mental health impacts been?
3. Where does your community get information regarding COVID-19 and the vaccines? a. Who are their trusted sources of information? Who are they skeptical of? b. What other information do they want to know about COVID-19 or the vaccine?
4. How do community members feel about the COVID-19 vaccines? a. Positive or negative? Trust it? Skeptical? Hesitant? i. Where do these feelings come from? b. Is it different for different groups? (e.g., old vs. young?)
5. What are the community's concerns regarding the vaccines? a. What previous concerns? How were they overcome? b. What are lingering/continuing concerns? c. What about boosters?
6. What may be preventing people in your community from getting the vaccine? a. Access? Fears? Don't think they are at risk? Concerns about side effects? Others?
7. How can we overcome these concerns and barriers for the community?
Now we are going to ask you some questions about COVID-19 clinical trials. *A clinical trial is a kind of research study. Clinical trials study if treatments or vaccines are safe for people and if they work like they are supposed to. Right now, clinical trials are being done across the U.S. to see if new treatments and vaccines for COVID-19 work to keep people healthy.*
8. Tell me about your experiences with clinical trials for the COVID-19 vaccine. a. Did you participate? b. Are you aware of anyone in your community participating in a clinical trial for the COVID-19 vaccine?
9. Are people in the community open/willing to participate in clinical trials? a. Why or why not? 10. What could motivate more people in your community to participate in clinical trials in the future?
11. More awareness of trials? A trusted source encouraging people? Incentives (money)? Understanding the benefits?

Focus group participants completed an online prefocus group survey that evaluated demographic information, access to care, vaccination status, COVID-19 risk, and clinical trial experiences. Each focus group lasted between 60 and 90 min via teleconference. Key informant interviews lasted between 40 and 75 min and were conducted in person or via teleconference. As an incentive, we offered a $50 gift card to all participants. This research was approved by the University of Houston IRB.

### Data analysis

Data analysis followed a thematic analysis approach. After each focus group or interview, C.N., L.G., and J.D. debriefed, reviewed the notes, and listened to the audio recording. Transcripts of English sessions and detailed notes of Vietnamese sessions were created and adjusted to accurately capture the conversations. C.N. and L.G. independently coded each session, using an inductive coding approach to derive preliminary codes directly from the data to reflect the participants' experiences. Analysis of data was ongoing, and C.N., L.G., and B.M.N. met regularly to discuss emerging codes and themes. Through this iterative group approach, a final set of codes and themes were collectively agreed upon by the research team using NVivo. These findings were then validated and refined based on feedback by the community partners BPSOSH and PIVOT. The resulting themes are representative of the repeated patterns of meanings throughout the interviews and focus groups.

## Results

**FIG. 1.**
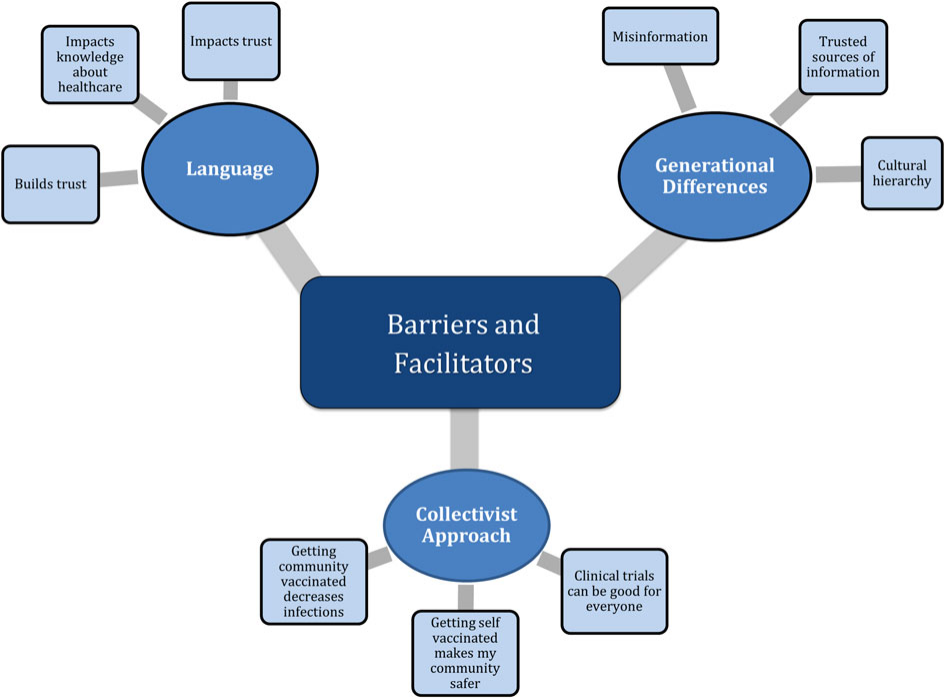
Conceptual Map of Thematic Analysis. *Blue ovals* represents themes; *Light blue boxes* represents subthemes.

In total, 20 adults participated in 4 focus groups with half held in Vietnamese. The sociodemographic characteristics of the focus group participants can be found in Table 3, while Table 4 provides information regarding participants' vaccination status and perceptions. For key informant interviews, two faith leaders, two health care workers, and one small business owner participated with three out of five interviews in Vietnamese. Table 5 presents the overall thematic analysis, including major themes, subthemes, and salient quotes from participants. Figure 1 displays how the themes intersect.

**Table 3.** Sociodemographic Characteristics of Focus Groups

Characteristic	Full sample (*N*=20)	
	*n*	***%***
Gender		
Female	15	75
Male	4	20
Prefer not to answer	1	5
Age, years		
18–25	11	55
26–40	2	10
41–56	2	10
57–75	5	25
Preferred language to use when discussing health care		
English	11	55
Vietnamese	9	45
Place of birth		
Born in the United States	9	45
Not born in the United States	10	50
Moved before the 1970s	0	0
Moved during 1970–1989	1	5
Moved during 1990–2009	1	5
Moved during 2010–2021	8	40
Prefer not to answer	1	5
Highest level of education		
Less than high school	4	20
High school or GED equivalent	5	25
Some college	7	35
Associate or college degree	3	15
Professional or doctorate degree	1	5

GED, General Educational Development.

**Table 4.** Prefocus Group Survey Results

Characteristic	Age	
	18–25 years (*N*=11)	26–75 years (*N*=9)
COVID-19 vaccine		
Have you received a COVID-19 vaccination?		
Yes	11	9
No	0	0
At what kind of place did you receive the most recent dose of your COVID-19 vaccine?		
Free standing retail store, for example, CVS, Walgreens	7	3
Health department clinic	0	2
Stadium	1	1
At workplace	0	1
Hospital	1	0
Family physician or other physician's office	0	1
University	1	0
To your knowledge, do you have or have you had COVID-19?		
No	7	5
Yes	2	4
I don't know	2	0
If yes, describe the level of care you received, or are receiving:		
Did not seek medical care	2	3
Received care, but was not hospitalized		1
How concerned are you about getting COVID-19? If you have already tested positive for COVID-19 or believe you have had COVID-19, answer based upon your risk for reinfection.		
Not at all concerned	1	1
A little concerned	2	3
Moderately concerned	5	2
Very concerned	3	3
How would you feel if you contracted COVID-19? If you have already tested positive for COVID-19 or believe you have had COVID-19, answer based upon how it made you feel.		
The worst thing that could happen	4	4
Very upset	5	0
Indifferent	0	1
A little upset	2	2
Not upset at all	2	0
How important is it for all people in your community to receive the COVID-19 vaccine?		
Very important	9	7
Important	2	2
Moderately important	0	0
Slightly important	0	0
Not important	0	0
Have you ever participated in a clinical trial?		
No, and I am not interested	2	5
No, but I am interested in learning more	8	4
Yes	1	0
I would be more interested in signing up for a clinical trial if… (select all that apply)		
I was confident that the trial would improve researchers' understanding of the disease	8	5
I had a trusted source of information about clinical trials	6	2
I had a better understanding of clinical trials, in general	6	2
I agreed with the goal of the clinical trial	4	2
The clinical trial did not cost my family anything	5	0
I felt the researchers had a good reputation	4	0
There was a clinical trial available within a 1-h drive	3	0
I knew another family enrolled in a clinical trial	1	2
My doctors suggested that a clinical trial might be a good fit	2	0
I have not signed up for a clinical trial because… (select all that apply)		
I may not like being in a clinical trial	5	4
I don't know about any clinical trials	5	3
The clinical trial may not be successful	5	1
I don't have enough information about the potential risks of clinical trials	5	0
I don't have enough information about the potential benefits of clinical trials	3	1
Being in a clinical trial would take too much time	2	2
For me, there may be more risks than benefits	3	0
I don't have enough information about the day-to-day requirements of clinical trials	2	1
A clinical trial would put a strain on my family	1	2
I could be hurt in a clinical trial	2	0
I am not interested in the kinds of treatments provided in clinical trials	2	0
The goals of clinical trials are not clear to me	1	1
Being in a clinical trial would interrupt my daily routine	1	1
I don't trust health care services or medical science in general	1	1
I don't trust the medical teams involved in clinical trials	1	0
Misinformation		
Have you seen or heard any information about COVID-19 vaccines (e.g., on the news, on social media, or from friends and family) that you could not determine was true or false?		
Yes	8	5
No	1	1
Not sure	2	3
How do you feel about the amount of information on COVID-19 vaccines that you are getting?		
I'm getting enough information	7	3
I'm not getting enough information	3	2
I'm getting too much information	1	4
Do you know where to get accurate, timely information about COVID-19 vaccines?		
Yes	8	5
No	2	3
Not sure	1	1
Select your top 3 most trusted sources of information about COVID-19 vaccines:		
CDC	9	4
U.S. government	2	5
Family and friends	2	3
FDA	3	2
Hospital system websites, for example, Memorial Hermann, Methodist	4	1
Local health officials	3	2
Primary care providers	2	2
Nurses	2	2
Social media, for example, Facebook, YouTube, Twitter, TikTok	0	2
News sources in English, for example, television, radio, internet	1	1
Professional organization(s), for example, American Medical Association	2	0
Pharmacists	1	0
State health departments	0	1
VietCOVID.org	1	0
News sources in Vietnamese, for example, television, radio, internet	0	1
Online publishers of medical information, for example, WebMD or Mayo Clinic	1	0
Viet Fact Check	0	1

CDC, Centers for Disease Control and Prevention; CVS, CVS Pharmacy, Inc; FDA, Food and Drug Administration.

**Table 5.** Themes and Subthemes for COVID-19 Vaccines and Clinical Trials from Focus Groups and Key Informant Interviews

Theme	Subthemes
Language	Language builds community Language impacts trust Language impacts knowledge about health care
Generational differences	Misinformation Trusted sources of information General information Participants 18–25 years of age Participants 45 years of age or older Cultural hierarchy
Collectivist approach and perspective	Getting the community vaccinated decreases infections Getting myself vaccinated makes my community safer Clinical trials can be good for everyone
Facilitators for vaccines	Family and friends Ease of access Privilege Trust in the vaccine Fear of hospitalization or death Financial Trusted sources of information
Barriers for vaccines	COVID-19 side effects Coverage Misinformation and lack of trust COVID-19 vaccines do not completely prevent getting COVID-19 Uncertainties with COVID-19 related to time COVID-19 vaccination stigma
Clinical trial barriers	Lack of time Lack of trust Lack of knowledge
Clinical trial facilitators	General thoughts Financial compensation Start with the younger generation Use trusted messenger

### Language

Participants reported limited availability of COVID-19 information in Vietnamese. They emphasized the importance of cultural competence and quality in translations to relay accurate information. In addition, well-translated health information in Vietnamese or a Vietnamese health care provider increases their trust in medicine. Participants also discussed how lack of translated materials likely prevents engagement with clinical trials.

### Generational differences

Many participants reported generational differences related to COVID-19 misinformation and trusted sources of information. Some individuals reported that language barriers impact older Vietnamese people's access to credible COVID-19 and vaccination information. In addition, younger participants reported cultural influences such as significance of familial hierarchy and respecting elders affecting discussions on COVID-19 misinformation.

### Collectivist approach and perspective

A collectivist perspective also played a role in receiving the vaccine or participating in clinical trials in the Vietnamese community. Participants identified the importance of increasing vaccination rates in the community to decrease COVID-19 infections and increase community safety as a facilitator of obtaining the vaccine. To increase clinical trial participation, individuals consistently stressed the potential of clinical trials to help the community.

### Facilitators for vaccines

Participants identified several facilitators to getting vaccinated. Many participants reported that the fear of severe illness or death from COVID-19 influenced their decision to get vaccinated. Many participants highlighted ease of access to get vaccinated in their communities. Other participants recognized the privilege in vaccine access in the United States compared with Vietnam. Overall, participants trusted the science behind the vaccine. They also revealed that their most trusted sources of information were medical professionals (especially of Vietnamese descent), faith-based organizations, and the government. Most participants conducted a cost–benefit analysis, and ultimately believed that the vaccine benefits outweighed the risks.

### Barriers for vaccines

Although all participants had received at least one dose of a vaccine, they still identified potential barriers. At the beginning of the pandemic, participants recalled inequalities in initial vaccine distribution, long wait times at vaccination centers, and initial fears related to misinformation about the efficacy and safety. Participants shared concerns that the vaccine would cause side effects or worsen their existing medical conditions. In addition, one participant reported a stigma of being vaccinated in their community.

### Clinical trials

Participants reported barriers that prevented clinical trial participation in their community: lack of trust in the government, time, and knowledge about clinical trials. Other participants suggested facilitators to increase interest and participation, such as increasing trust and awareness, and financial incentives. However, some participants did not believe any efforts would increase clinical trial participation due to cultural unfamiliarity.

## Discussion

A few overarching themes influencing participants' decision-making around COVID-19 vaccination and clinical trial participation emerged: language, generational differences, and a collectivist approach. Previous research has found a similar impact of collectivism to encourage optimal COVID-19 mitigation behavior.^8^ In studying Vietnamese Americans' willingness to vaccinate against COVID-19, risk of severe illness, ease of access, trust in science, and having trusted sources of information in culturally translated Vietnamese also arose as important facilitators. These facilitators of vaccination were also identified in studies focused on AANHPI populations.^9^ Participants reported barriers to vaccines such as concerns about vaccine safety, misinformation, and lack of health care access. Language barriers to COVID-19 information found in this study are consistent with previous research on ethnic minorities.^10^

Prior research demonstrated that AANHPI with limited English proficiency (LEP) were more likely to have unmet health care needs and communication problems in health care settings.^11,12^ Among the largest AANHPI groups in the United States, Vietnamese Americans have one of the highest rates of LEP with 48% of adults.^13^ Participants' concerns about trusted sources of information in Vietnamese echo challenges with misinformation in the Vietnamese community as well as other AANHPI populations.^14,15^ Our findings of uneasiness regarding language access to scientific evidence and importance of information dissemination through trusted messengers are consistent with prior qualitative research on COVID-19 vaccine acceptability in other populations.^10,16^

Apprehensions around clinical trial participation included lack of trust in the government, time, and knowledge and are similar to previous research on AANHPI populations.^17^ To increase therapeutic trial involvement, participants suggested underscoring the benefit of research on the community, raising awareness in Vietnamese, and providing monetary compensation. This is consistent with previous research that found focusing on altruistic messages from a physician of the same nationality and/or spoke the same language may motivate clinical trial participation in AANHPI.^18^ However, offering payment to research volunteers is a contentious practice.^19^

Our study is the first to examine Vietnamese Americans' perceptions of COVID-19 vaccination and clinical therapeutic trial participation. These findings can help public health departments and community-based organizations working with this population develop effective messages. However, this study contains limitations. The focus groups were held virtually so they did not include people with unreliable internet access. The sample size was small and did not contain unvaccinated individuals or folks living in rural areas due to limited funding and capacity. The few unvaccinated individuals recruited were unable to attend the focus groups. These results may not be replicable or generalizable. Nevertheless, the lack of COVID-19-specific studies involving and about Vietnamese Americans makes this a valuable contribution to the literature.

## Conclusion

In brief, culturally appropriate language, generational differences, and a collectivist approach were important themes impacting COVID-19 immunization and clinical trial participation in this Vietnamese American population. Promoting science-based information through trusted messengers, improving awareness and access, and illuminating benefits to the community may increase the uptake of COVID-19 vaccines and volunteering for therapeutic trials. These recommendations can help government agencies, health care providers, and community organizations working with Vietnamese American communities advance public health.

A potential next step is to survey a larger population of Vietnamese Americans to see if these results are replicable and to focus on COVID-19 vaccine facilitators and barriers among the unvaccinated Vietnamese American population. Based on comments from these participants, future COVID-19 research in Vietnamese Americans could explore mental health and the impact of rise in AANHPI hate crimes. Our community partners noted difficulties in recruiting unvaccinated individuals due to the need for separate focus groups to create a thoughtful permissive atmosphere. They also highlighted the slow uptake of COVID-19 vaccinations in children and boosters in adults along with difficulties in reaching rural, unhoused, or technologically limited populations.

## Abbreviations Used

AANHPI: Asian Americans, Native Hawaiians, and Pacific Islanders
BPSOSH: Boat People SOS Houston
CDC: Centers for Disease Control and Prevention
CVS: CVS Pharmacy, Inc
FDA: Food and Drug Administration
GED: General Educational Development
LEP: limited English proficiency
