# *Corrigendum to:* Identifying Facilitators and Barriers to Increasing COVID-19 Vaccination and Trial Participation in Vaccinated Vietnamese Americans

**DOI:** 10.1089/heq.2025.87633.correx

**Published:** 2025-06-27

Nguyen C, Gilbert L, Diep J, and Nguyen B-M. 2022. Identifying Facilitators and Barriers to Increasing COVID-19 Vaccination and Trial Participation in Vaccinated Vietnamese Americans. Health Equity 6: 485–493. doi: 10.1089/heq.2022.0032.

The funding information for the NIH agreement was not included in the original publication of this article. The online version has now been updated, and the funding information is also provided below.

